# Common neuromusculoskeletal injuries amongst rock climbers in the Western Cape

**DOI:** 10.4102/sajp.v71i1.227

**Published:** 2015-04-28

**Authors:** Liezel Wegner, Jarryd E. Pagel, Ashley W. Smit, Aimee Straszacker, Sarah L. Swart, St John Taft

**Affiliations:** 1 Department of Physiotherapy, University of the Western Cape, South Africa

## Abstract

**Background:**

Rock climbing is an extreme sport that is fast gaining interest in the Western Cape. Due to the physical nature of the sport, climbers often suffer neuromusculoskeletal (NMS) injuries. Physiotherapists are first-line practitioners who diagnose and treat NMS injuries, but no previous study has been conducted regarding common NMS injuries amongst rock climbers in the Western Cape.

**Objective:**

To determine the common NMS injuries amongst rock climbers, and the relationships between independent variables and injury.

**Method:**

A Quantitative, cross-sectional, retrospective descriptive study design utilised a self-developed survey based on the literature. This was completed by rock climbers from an indoor climbing gym in Cape Town and two outdoor crags in the Western Cape. Out of the total population of 650 climbers, 247 were conveniently sampled to complete the self-administered survey, making the results generalisable to the climbing population.

**Results:**

Finger flexor tendon pulley injuries were the most commonly diagnosed NMS injury. Injury to the fingers, hand and elbow regions were the most common self-reported injury by area. The risk of suffering climbing-related injuries was significantly correlated to gender, setting, grade and type of climbing, but not to frequency of climbing.

**Conclusion:**

The results of this study could assist physiotherapists to assess and manage the common NMS injuries that occur in this group of extreme athletes, as well as to raise awareness amongst rock climbers in the Western Cape about potential risk of injury.

## Introduction

Rock climbing is a rapidly growing extreme sport throughout the world (Pieber *et al*. 2012). In South Africa the Western Cape is a popular location for rock climbing, given its unique terrain and topographical features (Lourens 2005). Due to the physical intensity and nature of the sport, climbers are at risk of neuromusculoskeletal (NMS) injuries (Pieber *et al*. 2012). Physiotherapists assess and treat various sporting injuries, and have an important role to play in the prevention and rehabilitation of NMS injuries (World Confederation for Physical Therapy 2013).

In the most recent studies conducted by Mei-Dan and Carmont (2012) and Pieber *et al*. (2012), injury or rupture of the annular ligaments of the fingers or flexor pulley system is the most common NMS injury amongst rock climbers. In addition, elbow, forearm and wrist injuries are more common, according to an older article by Holtzhausen and Noakes (1996). These injuries included medial epicondylitis, brachialis tendonitis, biceps brachii tendonitis, ulnar collateral ligament sprain of the elbow, carpal tunnel syndrome, digital flexor tendon pulley sheath tears, interphalangeal joint effusions, fixed flexion deformities of the interphalangeal joints, and collateral ligament tears of the interphalangeal joints. Most frequent overuse syndromes identified in the literature amongst rock climbers were inflammation of the tendon sheaths and swelling of finger joints (Logan *et al*. 2004).The most common acute lower limb injuries amongst climbers are ankle fractures, sprains and strains as a result of falls to the ground (Limb 1995; Piebert *et al.* 2012). Types and risks of injuries can vary amongst different populations, and even though some international studies identify common injuries amongst rock climbers, thus far no similar study has been done in the South African context.

The aim of this study was to determine the common NMS injuries amongst rock climbers in the Western Cape, South Africa, and any relationships between independent variables and injury.

## Research design

### Research approach

This study utilised a quantitative approach, and a cross-sectional, retrospective descriptive design.

### Research method

#### Population and sampling

The study population consisted of members of City Rock indoor climbing club in Cape Town, as well as climbers approached at any of the crags within the Western Cape. The total climbing population identified accounted for 650 members. According to Yamane's formula (with an error variance of 5%) a sample size of 248 would make the findings of the study generalisable to the climbing population registered at clubs in the Western Cape. The final sample size obtained during data collection was 247. Participants were conveniently selected, and climbers participating in any form of the sport (traditional, bouldering or sport) for any length of time at any level were included. Data were only collected at City Rock on days when this was pre-arranged with the owners. Participants were recruited at the crags at random times that were convenient for the researchers.

#### Instrument for data collection

A self-administered survey questionnaire was developed based on similar studies in the literature (Jones, Ashgar & Llewellyn 2008; Limb 1995; Mei-Dan & Carmont 2012; Pieber *et al*. 2012), since no standardised rock climbing injury survey questionnaire currently exists. The survey questionnaire consisted of four sections, namely; demographic details, climbing information and experience, climbing injuries and medical care. For the purpose of this study the ‘time-loss’ definition of a sports injury was used, and the ‘time lost’ was specified as 2 weeks in order to exclude minor bruises and scrapes that could stop a climber from climbing for a day or two (Timpka *et al*. 2014).

#### Analysis of data

Data were coded and captured in Microsoft Excel and analysed using the Statistical Package for the Social Sciences (SPSS) version 22.0. Data were analysed for descriptive and inferential statistics. A univariate Chi-square test was used to test relationships between independent variables and injury, and the *p*-value for statistical significance was set at 0.005.

#### Validity

The newly developed survey was assessed for face and content validity by an expert in the field. Once recommended changes to the survey were made, a pilot study was conducted with 10 climbers in order to assess internal validity of the instrument. Participants were asked to comment on the difficulty of questions, and whether any of the questions seemed ambiguous (Peat *et al*. 2002). The participants in the pilot study reported that all of the questions were clear. No changes were made to the original survey after the pilot study, so the survey had good internal validity, and these 10 participants were included in the final study sample.

#### Reliability

A preliminary test-retest reliability study was performed with 11 participants in order to determine reliability of the individual constructs of the newly developed survey. According to Javali, Gudaganavar and Raj (2011) the ideal sample size for using Cronbach's alpha scores to determine definite reliability of a survey instrument is 50. Due to time constraints and since reliability testing of the survey was not the main aim of this study, recruiting 50 participants for retesting was not possible. The average Cronbach's alpha score for the individual questions of the survey (excluding demographic details) was 0.857 and the intra-class coefficient ranged from 0.148 to 1, with the confidence interval set at 95%. These results indicated good preliminary reliability and internal consistency of the different constructs of the survey. This finding is based only on preliminary reliability testing, and should be interpreted with caution. Further in-depth reliability testing of the instrument is recommended.

#### Generalisability

The results from this study would be generalisable to the rock climbing population in the Western Cape, since the sample is representative of the total population of climbers registered at the climbing gyms (Creswell, 2009).

#### Ethical considerations

Ethical clearance was obtained from the University of the Western Cape (Ref. 14/5/6) and permission was obtained from City Rock climbing gym telephonically to collect data. All participants were approached in person by the researchers, and written informed consent was obtained prior to any data being collected.

## Results

### Demographics

Of the total population (*N* = 247), 53% (*n* = 130) were male, 35% (*n* = 88) were female and 12% (*n* = 29) did not specify their gender. The mean age of the participants in this study was 26 years, and ages ranged from 15 to 68 years with a standard deviation of 12.8 years. The majority of the participants preferred sport climbing (44%), followed by bouldering (25%), whilst only 15% of this sample did traditional climbing. Sixteen per cent of the participants were unable to select which type of climbing they preferred, and did not complete the question.

### Climbing experience

The participants in this study were fairly evenly distributed and ranged from novice to very experienced climbers. Of the total population, 27% (*n* = 66) have been climbing for less than a year, 29% (*n* = 72) for 1–3 years, 15% (*n* = 38) for 3–5 years and 29% (*n* = 71) for more than 5 years. The climbing grades which participants reported ranged from grade 14 to grade 35, with a mean grade of 21 and a standard deviation of 6.

### Frequency of climbing

The frequency of climbing done by rock climbers varied from two or more times a week to less than once a month. The majority of participants in this study (64%; *n* = 157) climbed two or more times a week, 24% (*n* = 60) once a week, 5% (*n* = 11) twice a month, 0.4% (*n* = 1) once a month and 6.6% (*n* = 16) less than once a month.

### Climbing injuries

**FIGURE 1:**
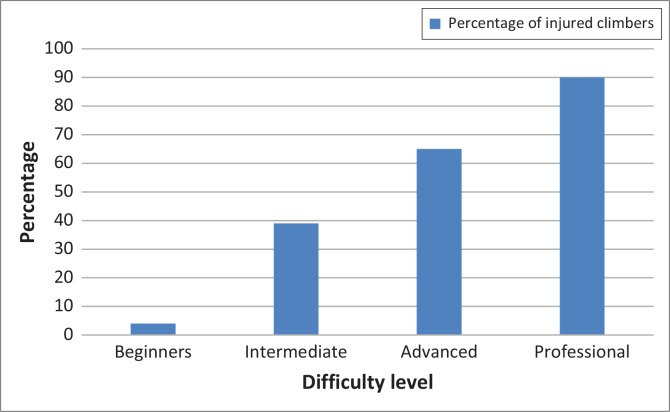
Percentage of injury according to difficulty of climbing.

The 247 participants suffered 371 injuries in the past year. Only 207 injuries were reported on in detail. The number of injuries suffered amongst the participants in this study ranged from no injuries to four injuries, with an average of 1.5 injuries per participant in the past year. The majority of injuries (57%) occurred during indoor climbing. Professional climbers suffered the most injuries (90%) relative to the difficulty of the grades climbed (Figure 1). The most common self-reported injuries by area and type are reported in Table 1. Table 2 shows the most common self-reported diagnoses and the number of participants that reported these.

**TABLE 1:** Most common self-reported injuries by area and type.

Area	Type of injury	% injured	*n*
Finger	Muscles or tendon/ ligament	19.8	41
Shoulder	Muscles or tendon/ ligament	9.7	20
Arm/elbow	Muscles or tendon/ ligament	8.2	17
Finger	Overuse	6.8	14
Back	Muscles or tendon/ ligament	4.8	10

*N* = 207.

**TABLE 2:** Most common self-reported diagnoses.

Diagnosed injury	% injured	*n*
Finger tendon	34.8	31
Elbow tendon	11.2	10
Shoulder muscle/tendon	7.9	7
Ankle ligament	6.7	6
Bicep tendon	4.5	4
Wrist tendon	4.5	4
Finger fracture	4,5	4
Shoulder dislocation	3.4	3
Ankle fracture	3.4	3
Rotator cuff	2.2	2

*N* = 89.

### Medical intervention

A minority of injured climbers (40%) sought medical intervention, and of those who sought medical intervention for their injuries, the majority (56%) sought physiotherapy. Other members of the health team who were approached included an acupuncturist, a chiropractor and an osteopath.

## Discussion

The majority of participants in this study had suffered an injury in the past year and 60% of these were muscle, tendon or ligament injuries. These findings are not unexpected or unusual; it is, however, important to understand and discuss the different findings in context to assist in meaningful interpretation.

The majority of the participants in this study (53%) were male. This finding was expected, but climbing seems to be gaining popularity amongst females when compared to older studies, which mostly reported on male participation (Jones *et al*. 2008).

In this study boulderers were more likely to suffer a climbing injury than sport or traditional climbers. Bouldering is the type of climbing with the least protective gear, therefore potentially leading to the higher risk of injuries whilst climbing. Bouldering also requires the most dynamic and explosive moves, whilst traditional and sport climbing are often slower and more controlled, possibly decreasing the risk of injury (Booth *et al*. 1999).

The high incidence of upper limb injuries amongst climbers in this study corresponds with international literature (Hochholzer & Schoffl 2003; Jones *et al*. 2008; Logan *et al*. 2004). The increased demands placed on the upper limbs during climbing predispose the upper limbs to NMS injuries. The area and type of injuries reported on in this study were mostly finger, elbow and shoulder muscle tendon or ligament injuries. This finding could be due to the nature of the sport that loads the fingers in different positions. This finding correlates with the literature stating that the most common upper limb injury sustained by rock climbers is finger pulley injuries (Logan *et al*. 2004; Mei-Dan & Carmont 2012; Tator 2008).

The high number (90%) of professional climbers who sustained injuries is alarming. This finding is contrary to the intuitive expectation that beginner climbers would be injured more frequently. However, this finding does correlate with the findings of Jones *et al*. (2008), who also reported a higher prevalence of injuries amongst climbers who climbed higher grades and more technical routes. Climbing higher grades predisposes climbers to injury as the more technical routes require longer run-outs (distances between protective gear placements), and higher force demands.

In this study climbers were more frequently injured whilst climbing indoors as opposed to outdoors. This could be due to the fact that climbers take more risks when climbing indoors as they feel more protected and feel less likely to get injured. The literature suggests that risk-taking behaviour may be modified to account for the availability of safety equipment at indoor climbing walls. However, according to Limb (1995) indoor climbing walls seem to be associated with a very low injury rate.

A relatively small percentage of injured climbers in this study sought medical intervention for their injuries. However, the majority of climbers sought physiotherapy intervention for their injuries. Climbers seem to be reluctant to seek medical intervention, but when they do they are aware of physiotherapy services. The question is how many physiotherapists are aware of the common NMS injuries that are suffered by this unique group of athletes?

### Limitations of the study

A common limitation with a retrospective self-reported injury survey is that it is subject to recall bias. Participants might not be able to accurately recall the details of an injury sustained a few months ago. The findings of the study might also be biased towards the profile of indoor rock climbers, since a large proportion of the participants were recruited from an indoor climbing gym. However, most rock climbers use an indoor gym for training to climb outdoors. Based on the findings of this study future research should be conducted on prevention and treatment of finger tendon injuries amongst rock climbers.

### Recommendations

This study provides baseline information on the common NMS injuries sustained by rock climbers in the Western Cape and the profile of climbers who are more likely to suffer injury. Injured rock climbers most commonly seek physiotherapy intervention for their injuries, and it is recommended that therapists treating rock climbers understand the physiological demands required by the sport in order to formulate strategies for prevention of finger tendon injuries. Rock climbers who climb harder grades, and often do indoor bouldering should also be aware of their accumulative risk for finger injury and take appropriate precautionary measures, such as finger strapping.

## Conclusion

The literature on the topic of rock-climbing injuries is scarce and in many instances outdated. This study aimed to describe the common NMS injuries suffered by rock climbers in the Western Cape region of South Africa. The findings of this study could potentially assist physiotherapists with a special interest in sports injuries to raise awareness about the common injuries suffered by this fast-growing group of extreme athletes. It could also assist in injury prevention amongst the rock climbing community as well as aiding in the understanding of the specific types of injuries occurring within this specific sport.

## References

[CIT0001] Booth J., Marino F., Hill C. & Gwinn T., 1999, ‘Energy cost of sport rock climbing in elite performers’, *British Journal of Sports Medicine* 33, 14–18. 10.1136/bjsm.33.1.14 10027051 PMC1756138

[CIT0002] Creswell J.W., 2009, *Research Design Qualitative, quantitative and mixed methods approaches*, Sage Publications, Thousand Oaks, CA.

[CIT0003] Hochholzer T. & Schoffl V., 2003, *One Move Too Many*, Lochner-Verlag Ebenhausen, Germany.

[CIT0004] Holtzhausen L.M. & Noakes T.D., 1996, ‘Elbow, forearm, wrist, and hand injuries among sport rock climbers’, *Clinical Journal of Sports Medicine* 6(3), 196–203. 10.1097/00042752-199607000-00010 8792052

[CIT0005] Javali S.B., Gudaganavar N.V. & Raj S.M., 2011, ‘Effect of varying sample size in estimation of coefficients of internal consistency’, *Webmed Central Biostatistics*, 2(2):WMC001649, viewed 11 November 2014, from http://www.webmedcentral.com/wmcpdf/Article_WMC001649.pdf

[CIT0006] Jones G., Ashgar J. & Llewellyn D.J., 2008, ‘The epidemiology of rock-climbing injuries’, *British Journal of Sports Medicine* 42(9), 773–778. 10.1136/bjsm.2007.037978 18065444

[CIT0007] Limb D., 1995, ‘Injuries on British climbing walls’, *British Journal of Sports Medicine* 29(3), 168–170. 10.1136/bjsm.29.3.168 8800849 PMC1332307

[CIT0008] Logan A.J., Makwana N., Mason G. & Dias J., 2004, ‘Acute hand and wrist injuries in experienced rock climbers’, *British Journal of Sports Medicine* 38, 545–548. 10.1136/bjsm.2002.003558 15388536 PMC1724952

[CIT0009] Lourens T., 2005, *Western Cape Rock*, Blue Mountain Design & Publishing, Cape Town.

[CIT0010] Mei-Dan O. & Carmont M., 2012, *Adventure and extreme sports injuries: Epidemiology, treatment, rehabilitation and prevention*, Springer-Verlag, London.

[CIT0011] Neumann D.A., 2002, Kinesiology of the musculoskeletal system, Mosby, St Louis.

[CIT0012] Peat J., Mellis C., Williams K. & Xuan W., 2002, *Health science research: A handbook of quantitative methods*, Sage, London.

[CIT0013] Pieber K., Angelmaier L., Csapo R. & Herceg M., 2012, ‘Acute injuries and overuse syndromes in sport climbing and bouldering in Austria: A descriptive epidemiological study’, *Central European Journal of Medicine* 124(11–12), 357–362. 10.1007/s00508-012-0174-5 22661041

[CIT0014] Tator C.H. (ed.), 2008, *Catastrophic injuries in sports and rehabilitation: Causes and prevention: A Canadian study*, University of Toronto Press, Toronto.

[CIT0015] Timpka T., Alonso J.M., Jacobsson J., Junge A., Branco P., Clarsen B. et al, 2014, ‘Injury and illness definitions and data collection procedures for use in epidemiological studies in Athletics (track and field): Consensus statement’, *British Journal of Sports Medicine* 48, 483–490. 10.1136/bjsports-2013-093241 24620036

[CIT0016] World Confederation for Physical Therapy, 2013, ‘What is Physiotherapy?’, viewed 14 October 2014, from http://www.wcpt.org

